# The effects of inflammatory bowel disease on caregivers: significant burden and loss of productivity

**DOI:** 10.1186/s12913-020-05425-w

**Published:** 2020-06-18

**Authors:** Aria Zand, Brian J. Kim, Welmoed K. van Deen, Zachary Stokes, Anya Platt, Shelby O’Hara, Harrison Khong, Daniel W. Hommes

**Affiliations:** 1 UCLA Center for Inflammatory Bowel Diseases, Vatche and Tamar Manoukian Division of Digestive Disease, David Geffen School of Medicine, University of California at Los Angeles, 10945 Le Conte Ave #2338, Los Angeles, CA 90095 USA; 2 Department of Digestive Diseases, Leiden University Medical Center, Leiden, the Netherlands; 3 Cedars-Sinai Center for Outcomes Research and Education, Division of Health Services Research, Cedars-Sinai Medical Center, California, Los Angeles USA

**Keywords:** Caregiver, Productivity, Quality of life, Caregiver burden

## Abstract

**Background:**

Caregiver burden is the emotional, physical, practical, and/or financial burden associated with taking care of a patient with a chronic condition. Limited literature on caregiver burden in Inflammatory Bowel Diseases (IBD) has accounted for some predictors, but its effect on work productivity (absenteeism and presenteeism) is unknown.

**Methods:**

In a prospective study, patients and their respective caregivers were surveyed from November 2015 until July 2017. Data on demographics, work productivity, quality of life, disease activity, caregiver burden and productivity were collected. The burden on caregivers was assessed and associations between caregiver productivity and caregiver burden were analyzed. Additionally, predictors for caregiver burden were identified.

**Results:**

One hundred two IBD patients and their respective caregiver were included. In total, 39% of IBD caregivers experienced burden. Caregivers with burden experienced significantly more absenteeism and presenteeism (65 and 85% respectively). Furthermore, 51% of caregivers felt that they should be doing more for their care recipient and felt they could do a better job at caregiving. Predictors of burden included race/ethnicity, history of fistulas, diagnosis of ulcerative colitis, higher caregiver education, and hours spent caregiving.

**Conclusion:**

Caregivers with burden had significantly more productivity decrease compared to those without burden. Additionally, the majority of caregivers feel they should be providing more and better care for their recipients. The development of strategies to address caregiver’s distress and perceived burden when caring for IBD patients is warranted.

## Background

**Figure 1:**
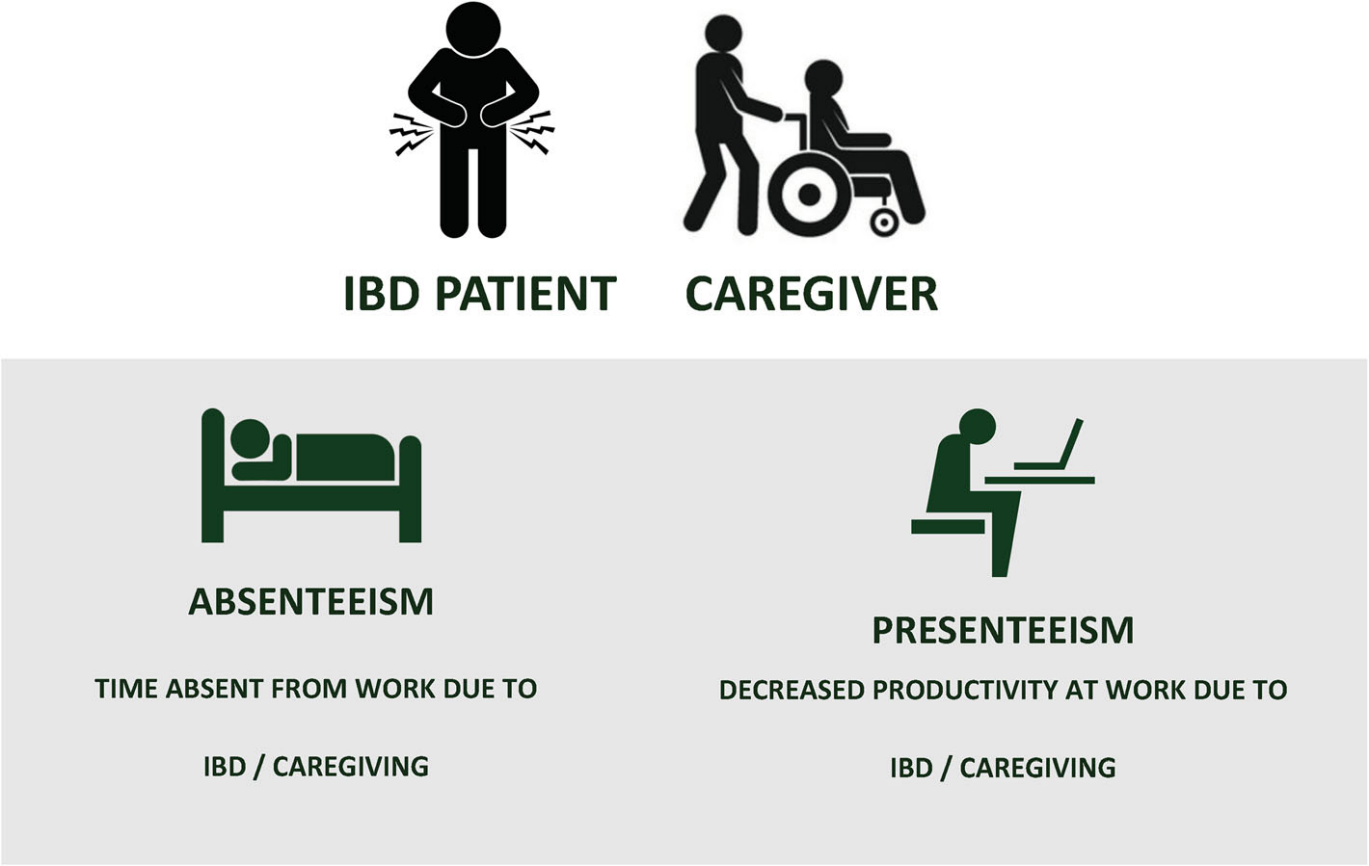
**Fig. 1** Absenteeism and presenteeism in IBD patients and their respective caregivers

Inflammatory Bowel Diseases (IBD), such as Crohn’s disease (CD) and ulcerative colitis (UC), are chronic immunological digestive diseases generally characterized by frequent abdominal pain and diarrhea with the disease state alternating between remission and exacerbation [1]. IBD affects nearly 3 million Americans who frequently require medical therapy, surgeries, and hospitalizations [2]. A study performed by Lönnfors et al. [3]. among 4670 IBD patients from 25 countries found that 22% of IBD patients experienced periodic flare-ups. During a flare-up, 38% spent days in the hospital, 62% experienced gastrointestinal bleeding, and 87% experienced abdominal pain at least once a week. Furthermore, their study showed that a third of IBD patients felt their intimate relationships were compromised, a quarter of IBD patients felt it is difficult to maintain friends, 67% was concerned about the availability of toilets when planning to attend an event, and 40% woke up frequently due pain associated with their IBD In the workplace, IBD patients reported fatigue, irritability, and demotivation. Additionally, IBD patients had difficulty coping with IBD-related limitations in the workplace resulting in increased stress-levels, lower quality of life (QoL) and a higher likelihood of absenteeism (time missed from work due to disease) and presenteeism (being present at work, but less productive due to disease), see Fig. 1 [4].

The high strain of IBD is not limited to patients but also impacts their caregivers. Caregiver burden is described as the emotional, physical, practical, and/or financial burden associated with taking care of a patient with a chronic condition. An informal caregiver, usually a family member or spouse, aids the care-recipient with their medication, post-operative wound dressing, and transport to the clinic. Especially when the state of the disease fluctuates between remission and exacerbation, the caregiver has to respond to the unpredictable demands of the disease. Several studies have brought caregiver burden in IBD to light. Gray et al. found that pediatric IBD patients’ disease activity increased parental stress [5]. Akobeng et al. showed that the source of parental anxiety and stress is largely due to concerns about the effects that IBD might have on their child’s future [6]. A study by Parekh et al. in adult IBD patients found that caregiver burden is frequent in this population as well, affecting 44% of caregivers. Factors such as the presence of another dependent in the home (aside of the patient), the disease severity, and a caregiver’s history of psychiatric illness were found to be predictors for caregiver burden and low QoL [7].

A more recent review by Shukla et al. reiterates the current scarcity of literature on caregiver burden in IBD and the lack of interventions that address caregiver burden [8]. Although the literature on IBD caregiver burden is limited, studies that assess the QoL of caregivers and the effects of caregiving for patients with other chronic conditions exist. Baanders and Heijmans reported that 53% of partners of those diagnosed with a chronic condition found that the chronic condition of their loved one put a strain on their personal life, while other partners reported personal burden, changes in their social relations, and financial nuisances [9]. Caregivers were reported to develop mental distress (e.g. depression, anxiety), found to use significantly more healthcare resources (i.e. physician and emergency visits), and in the case of elderly spouses, 63% higher mortality than non-caregivers [10, 11]. Hours spent caregiving correlated with a decrease in work productivity and physical activity [12].

An caregiver’s burden can easily go unnoticed. In order to develop effective interventions to relieve caregiver burden, it is imperative to obtain an in-depth understanding of the physical, mental, and social consequences of caregiving. More information is needed about the causes and consequences of caregiver burden in IBD, including the effects on work productivity*.* The aim of this study was to investigate the burden of IBD on caregivers, their work productivity (in terms of absenteeism and presenteeism), and to identify patient characteristics associated with caregivers’ outcomes.

## Methods

### Objectives

The primary study objective was to investigate the impact of IBD on informal caregivers and to identify predictors for caregiver burden. The secondary objective was to assess the association between caregiver burden and QoL, activity impairment and work productivity in IBD patients and caregivers.

### Design and population

For this cross-sectional study, IBD patients had to be at least 18 years old and to be diagnosed with UC or CD confirmed by endoscopy or radiology evaluation. Caregivers were informal, had to be at least 18 years old and had to assist an IBD patient with managing and/or coping with their disease, for instance by assisting them with post-operative wound dressing, helping with medications, and/or accompanying patients to the clinic. All participating IBD patients and caregivers consented to participate.

All patients enrolled in the UCLA Center for Inflammatory Bowel Diseases were approached via email to participate in a survey from November 2015 until September 2016. Additionally, patients and caregivers were asked to participate in person to participate between September 2016 and November 2017 during outpatient clinic visits. Through email, patients were sent an IBD patient survey and were asked to forward the caregiver survey to their respective caregiver. In clinic, IBD patients and caregivers filled out the survey on a tablet. If they were unable to finish, they were provided with a link to finish the survey at home. REDCap (Research Electronic Data Capture) was used to host a de-identified web-based questionnaire accessible through a 128-bit SSL encrypted link [13]. Both patient and caregiver were given a unique matching subject ID to confirm that both IBD patient and caregiver completed their respective surveys and to match the survey results to each other.

### Questionnaires & Definitions

Two types of surveys were administered, one for the IBD patient and one for the caregiver. The questionnaires used for the IBD patient included: 1) basic demographics, 2) the Work Productivity and Activity Impairment Questionnaire for IBD (WPAI-IBD), which measures absenteeism (the time absent from work due to IBD) and presenteeism (decreased productivity at work due to IBD) [14], 3) the short-IBD Questionnaire (sIBDQ) to measure QoL [15]; the sIBDQ score ranges from 10 (worst QoL) to 70 (best QoL), and 4) the mobile Health Index UC (mHI-UC) or CD (mHI-CD) [16], a validated questionnaire to assess disease activity remotely.

The questionnaires used for the caregiver included: 1) basic demographics, 2) the Work Productivity and Activity Impairment Questionnaire for caregivers (WPAI-CG), which measures absenteeism (the time absent from work due to caregiving) and presenteeism (decreased productivity at work due to caregiving) [14], and 3) the Zarit Burden Interview Score (ZBI), a set of 22 questions that determine a caregiver’s burden, and which categorizes caregiver burden in 4 levels: 1. Little or no burden, 2. Mild to moderate burden, 3. Moderate to severe burden, 4. Severe burden [17].

### Statistical analysis

Descriptive statistics were provided for the result of the questionnaires. The two-sided Fisher’s exact test was used to test for associations between categorical variables, the Student’s t-test was used to compare means between groups. Patients with two caregivers were analyzed twice as separate patients.

A simple logistic regression model was used to examine which IBD patient and caregiver features predict caregiver burden. Caregiver burden was defined as *any* caregiver burden as indicated by ZBI levels 2–4 (mild – severe burden). Caregiver’s demographics (i.e. age, gender, relationship to patient, education level, annual income, duration of caregiving, etc.) and IBD patient’s characteristics (i.e. demographics, IBD type, QoL, productivity, etc.) were included in the model as independent variables.

All variables with p-value ≤.35 in the simple logistic regression analysis were subsequently included in a multiple logistic regression model to assess their independent contribution to caregiver burden. A backward selection model was run in which non-significant variables (*p* > .05) are removed in a step-wise fashion until only significant predicators of caregiver burden (*p* < .05) remained.

Statistical analyses were performed using statistical package program R 3.4.0 [18].

### Ethical considerations

The study was approved by the University of California Los Angeles Institutional Review Board (UCLA IRB) protocol number 15–001304. All subjects gave their informed consent before entering the study.

## Results

**Figure 2:**
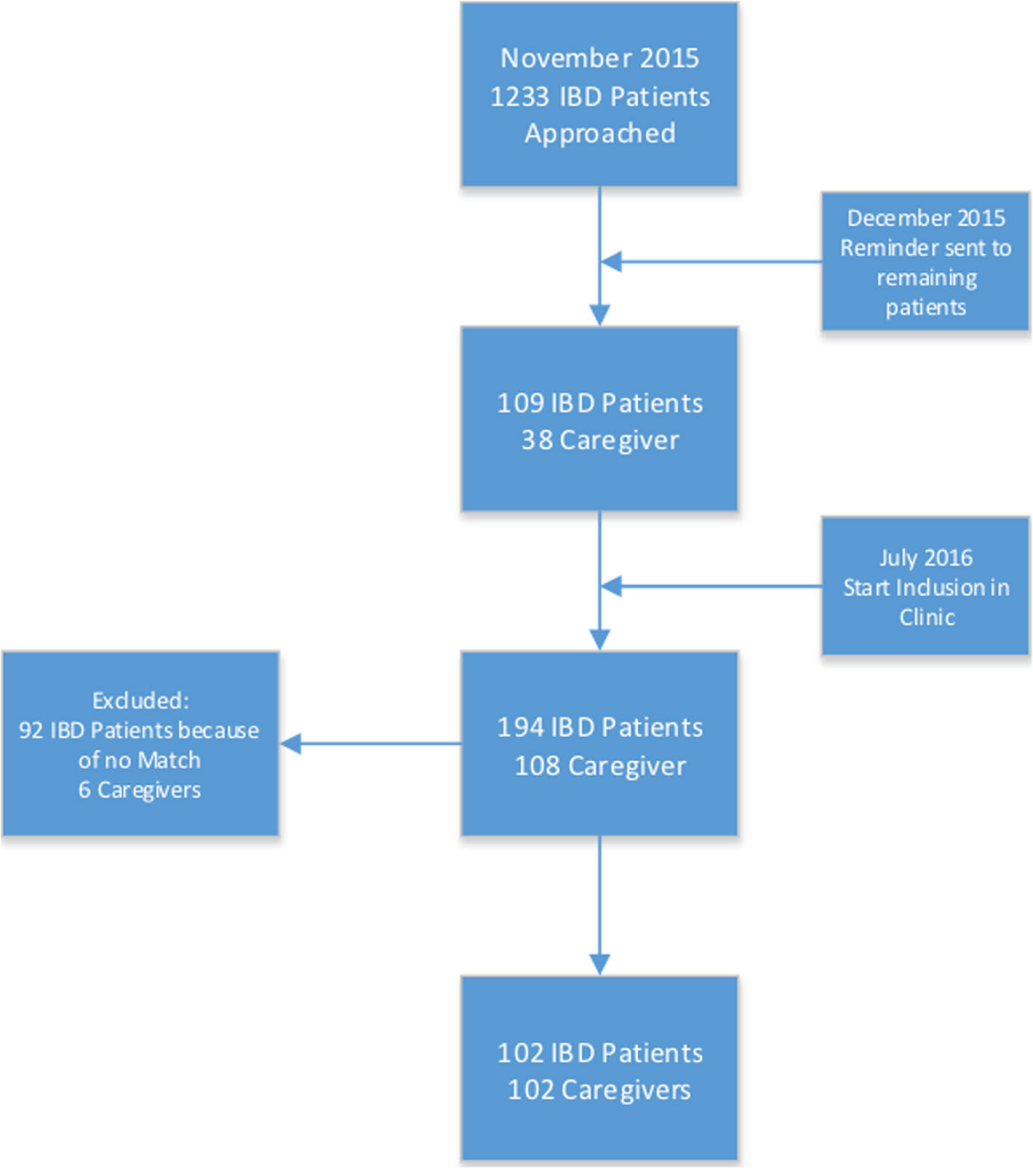
**Fig. 2** Flowchart of study inclusion

In November 2015, 1233 patients of the UCLA Center of Inflammatory Bowel Diseases and their respective caregiver(s) were invited to participate in the online survey, an additional reminder was sent in December 2015. In total 109 IBD patients and 38 matching caregivers responded. In order to increase the study population, from July 2016 to November 2017 we included additional patients and caregivers in the clinic of our tertiary IBD center. This led to a total cohort 194 IBD patients and 108 caregivers. We excluded 92 IBD patients because we did not have a matching caregiver and 6 caregivers were excluded because of erroneous entry (e.g. did not finish survey or incorrect entry of data); 2 patients indicated having two caregivers. This resulted in a final cohort of 102 IBD patients and 102 matching caregivers (Fig. 2).

The 102 IBD patients who were successfully matched to a caregiver were more frequently female (*p* < 0.01), were older (*P* = 0.02), had fewer non-Hispanic whites (*p* = 0.02), fewer surgeries (*p* = 0.01), less active disease (*p* < 0.01), lower employment rates (*p* < 0.01) and less activity impairment (*p* = 0.01) than the 92 IBD patients that were not successfully matched to a caregiver (Supplementary Table 1).

### Patient population

Table 1 summarizes the characteristics of the enrolled patients and their caregivers. Fifty-two percent were diagnosed with CD (*n* = 53) and 48% with UC (*n* = 49); 49% of patients had active disease as defined by the mHI-CD or mHI-UC at the time of the survey. There was no significant difference in the prevalence of disease activity between UC and CD patients (*p* = 0.07). The mean age was 39 years (SD 16), 70% were female (*n* = 71), and 60% (*n* = 60) were of white non-Hispanic origin. In total, 16% (*n* = 16) of CD patients and 6% (*n* = 6) of UC patients were taking biologics; 11% (*n* = 28) of CD patients and 15% (*n* = 15) of UC patients were on a combination of two or more medications; 18% (*n* = 18) of CD patients and 7% (*n* = 7) of UC patients indicated not to use any IBD-related medication.

**Table 1:** The characteristics of IBD patients and Caregivers

Variable	CD (***n*** = 53)	UC (***n*** = 49)	Caregivers (***n*** = 102)
**Age, mean (SD)**	37.7 (17.1)	40.9 (15)	48 (15.5)
**Gender % (n)**	69.8% Female (37)	69.4% Female (34)	48% Female (49)
**Race % (n)**			
White Non-Hispanic	67.9% (36)	71.4% (35)	58.8% (60)
White Hispanic	11.3% (6)	12.2% (6)	13.7% (14)
Other	5.6% (3)	8.2% (4)	11.8% (12)
Black/African American	5.6% (3)	0% (0)	3.9% (4)
Asian	7.5% (4)	8.2% (4)	11.7% (12)
American Indian/Alaska Native	1.9% (1)	0% (0)	0% (0)
**Abdominal Surgery % (n)**	52.8% Yes (28)	12.2% Yes (6)	N/A
**Fistula % (n)**	41.5% Yes (22)	16.3% Yes (8)	N/A
**Medication Use % (n)**			N/A
Biologics	16% (16)	6% (6)	
5ASA	2% (2)	11% (11)	
Immunomodulators	5% (5)	1% (1)	
Steroids	2% (2)	4% (4)	
Others (Antibiotic, Antispasmodic, Anti-diarrheal)	0% (0)	2% (2)	
Combo	11% (11)	15% (15)	
No IBD Related Medication	18% (18)	7% (7)	
**Disease State (mHI) % (n)**	58.5% Active Disease (31)	38.8% Active Disease (19)	N/A
**Disease Location**	26.4% (14) Small Bowel	2.0% (1) Proctitis	
	17.0% (9) Large Bowel	69.4% (34) Pancolitis	
	37.7% (20) Both	12.2% (6) Left-sided	
	18.9% (10) Unknown	16.3% (8) Unknown	
**Disease Duration in years, mean (SD)**	14.2 (9.8)	16.8 (18.6)	N/A
**Quality of Life, mean (SD)**	44.4 (12.2)	47.3 (13.1)	N/A
**Employed % (n)**	43.4% Yes (23)	59.2% Yes (29)	71.6% Yes (73)
**Of those employed:**	Due to IBD	Due to IBD	Due to IBD caregiving
**Absenteeism (Yes/No) last week % (n)**	52.2% Yes (12)	27.6% Yes (8)	38.4% Yes (28)
**If yes, mean absenteeism hours %**	15%	7.1%	9.1%
**Of those employed:**	Due to IBD	Due to IBD	Due to IBD caregiving
**Presenteeism (Likert) % (n)**	78.3% Yes (18)	58.6% Yes (17)	57.5% Yes (42)
**Mean Presenteeism %**	30.6%	27.3%	21.5%
**For the entire group:**	Due to IBD	Due to IBD	Due to IBD caregiving
**Activity Impairment (Likert) % (n)**	84.9% Yes (45)	71.4% Yes (35)	52% Yes (53)
**Mean Activity Impairment %**	38.9%	36.3%	18.7%
**Relationship to Patient % (n)**	N/A	N/A	55.9% Spouse or Partner (57)
			23.5% Parent/Family member (24)
			13.7% Child (14)
			6.9% Other (7)
**Environment % (n)**	N/A	N/A	74.5% Living with Patient (76)
			25.5% Living separately of Patient (26)
**Education Level % (n)**	N/A	N/A	77.5% College or Post-College Degree (79)
			22.5% College-degree or less (23)
**Annual Income level % (n)**	N/A	N/A	47.1% $100,000 or more (48)
			52.9% Less than $100,000 (54)
**Mean Time Spent Caregiving** **(hours/week SD)**	N/A	N/A	12.2 h (25.4 h)
**Mean Duration of Caregiving (SD)**	N/A	N/A	8.1 years (8.5)
**Chronic Disease % (n)**	N/A	N/A	13.7% Yes (14)

In total 50% of IBD patients were employed, of whom 39% (*n* = 20) experienced absenteeism within the last week, with a mean of 10% of work hours missed (SD 20%); 66% experienced presenteeism with a mean decrease of 27% in productivity at work (SD 31%). The mean QoL, measured by the sIBDQ, was 45 (SD 13; Table 1).

### Caregiver population

The mean age of the caregivers was 48 years, 48% were female (*n* = 49), and 59% (*n* = 60) were of white non-Hispanic origin. In total, 56% of caregivers were a spouse or partner, 24% were a parent or a other family member, 14% were a child of the patient and 7% were in another category. Furthermore, we found that 75% (*n* = 76) of caregivers lived with the IBD patient, whereas 25% (*n* = 26) did not. The caregivers spent an average of 12 h (SD 25) per week on caregiving and had been caregiving for an average of 8.1 years (SD 8.5). In total 13.7% of caregivers indicated that they suffered from a chronic disease themselves (Table 1).

The majority 77% (*n* = 79) had finished college or post college and 47% had an income of $100,000 or more. The employment rate in the caregiver population was 72% (*n* = 73), of whom 38% (*n* = 28) experienced absenteeism within the last week, with a mean of 9% of work hours missed (SD 17%); 57% experienced presenteeism with a mean decrease of 22% in productivity at work (SD 30%).

### Caregiver burden

Using the ZBI, we found that 39% (*n* = 40) of caregivers experienced caregiver burden (either mild, moderate or severe). IBD caregivers were impacted by caregiving because they felt stressed between caring for the care recipient and trying to meet other responsibilities for family or work (41%), they experienced fear for the future of the care recipient (73%) or felt that their caregiver was dependent on them (55%). Additionally, 51% of caregivers felt that they should be doing more for their care recipient and felt they could do a better job at caregiving (Table 2). Importantly, 32% felt uncertain about what to do with their care recipient (question 19).

**Table 2:** Burden on Caregivers as measured by the ZBI

Zarit Burden Interview Results Among Caregivers						
Question	Never	Rarely	Sometimes	Quite Frequently	Frequently	Nearly Always
**1. Do you feel that your care recipient asks for more help than he/she needs?**	65%	24%	10%	2%	0%	0%
**2. Do you feel that because of the time you spend with your care recipient that you don’t have enough time for yourself?**	51%	17%	26%	3%	0%	3%
**3. Do you feel stressed between caring for your care recipient and trying to meet other responsibilities for your family or work?**	30%	28%	30%	6%	0%	5%
**4. Do you feel embarrassed over your care recipient behavior?**	73%	16%	12%	0%	0%	0%
**5. Do you feel angry when you are around your care recipient?**	68%	23%	9%	0%	0%	1%
**6. Do you feel that your care recipient currently affects your relationships with other family members or friends in a negative way?**	62%	22%	14%	2%	0%	1%
**7. Are you afraid what the future holds for your care recipient?**	14%	14%	40%	22%	1%	10%
**8. Do you feel your care recipient is dependent on you?**	15%	30%	37%	13%	1%	4%
**9. Do you feel strained when you are around your care recipient?**	51%	25%	23%	1%	0%	1%
**10. Do you feel your health has suffered because of your involvement with your care recipient?**	67%	16%	14%	4%	0%	0%
**11. Do you feel that you don’t have as much privacy as you would like because of your care recipient?**	73%	16%	7%	3%	0%	2%
**12. Do you feel that your social life has suffered because you are caring for your care recipient?**	50%	24%	22%	3%	0%	2%
**13. Do you feel uncomfortable about having friends over because of your care recipient?**	81%	9%	9%	0%	0%	1%
**14. Do you feel that your care recipient seems to expect you to take care of him/her as if you were the only one he/she could depend on?**	54%	20%	16%	6%	0%	5%
**15. Do you feel that you don’t have enough money to take care of your care recipient in addition to the rest of your expenses?**	51%	17%	24%	5%	1%	3%
**16. Do you feel that you will be unable to take care of your care recipient much longer?**	80%	12%	6%	2%	0%	0%
**17. Do you feel you have lost control of your life since your care recipient’s illness?**	71%	11%	16%	3%	0%	0%
**18. Do you wish you could leave the care of your care recipient to someone else?**	75%	14%	9%	1%	0%	1%
**19. Do you feel uncertain about what to do about your care recipient?**	37%	30%	25%	6%	0%	1%
**20. Do you feel you should be doing more for your care recipient?**	25%	25%	35%	13%	0%	3%
**21. Do you feel you could do a better job in caring for your care recipient?**	22%	27%	38%	11%	0%	2%
**22. Overall, how burdened do you feel in caring for your care recipient?**	46%	35%	11%	7%	0%	1%

### Predictors of caregiver burden

We explored if caregiver burden had an association with absenteeism, presenteeism and activity impairment in the IBD and caregiver population. We also looked at the association between caregiver burden and the IBD patients’ and caregiver characteristics. We found that patients with lower QoL (*p* = .04), more absenteeism (*p* = .03), more presenteeism (*p* < .01) or more activity impairment (*p* < .01) were more likely to have a caregiver who experiences burden. The age of the patient and the caregiver relationship were not associated with caregiver burden. More importantly, caregivers who experienced burden had significantly more absenteeism (*p* = .04), presenteeism (*p* < .01) and activity impairment (*p* < .01) themselves than caregivers who did not experience caregiver burden (Table 3).

**Table 3:** Comparison of IBD population with and without caregiver burden. Univariate logistic regression models for differences * = *P* < .35, # = not considered, bold = *P* < .05

IBD Population (***n*** = 102)	Caregiver Burden 39% (40)	No Caregiver Burden 61% (62)	***p***-value
**Burden Type**	82.5% (33) Mild to Moderate		N/A
	15% (6) Moderate to Severe		
	2.5% (1) Severe		
**IBD patient characteristics**			
Age mean (SD)	41.3 (16.8)	37.9 (15.6)	*P = 0.30
Gender % (n)	62.5% Female (25)	74.2% Female (46)	*P = 0.21
Race % (n)	75% White Non-Hispanic (30)	66.1% White Non-Hispanic (41)	*P = 0.34
	25% Other (10)	33.9% Other (21)	
Abdominal Surgery % (n)	30% (12)	35.5% (22)	*P* = 0.57
Fistula % (n)	35% (14)	25.8% (16)	*P = 0.32
Disease type % (n)	55% UC (22)	43.5% UC (27)	*P = 0.26
Disease State (mHI) % (n)	65% Active (26)	38.7% Active (24)	*P **= 0.01**
IBD Quality of Life, mean (SD)	39.85 (12.29)	46.12 (11.68)	*P **< 0.01**
Employed % (n)	50% Yes (20)	52% Yes (32)	#*P* = 0.24
IBD Absenteeism % (n)	65% Yes (13)	21.9% Yes (7)	*P **< 0.01**
Mean Absenteeism % (SD)	19% (11.9)	5% (12.4)	**#*P*** **= 0.03**
IBD Presenteeism % (n)	85% Yes (17)	56.3% Yes (18)	*P = 0.05
Mean Presenteeism % (SD)	45.2% (35.6%)	14.5% (19.5)	# *P* < 0.01
Activity Impairment % (n)	87.5% Yes (35)	72.6% Yes (45)	*P = 0.08
Mean Activity Impairment % (SD)	52.3% (31.1)	28.2% (27.9)	**#*P*** **< 0.01**
**Caregiver characteristics**			
Caregiver age mean (SD)	47.3 (14.2)	48.5 (16.3)	*P* = 0.71
Caregiver gender % (n)	50% Female (20)	46.8% Female (29)	*P* = 0.75
Living together % (n)	22.5% No (9)	27.4% No (17)	*P* = 0.58
Caregiver relationship % (n)	52.5% Spouse/Partner (21)	58.1% Spouse/Partner (36)	*P* = 0.58
Caregiver education % (n)	82.5% College or Post-College (33)	74.2% College or Post-College (46)	*P = 0.33
Caregiver income % (n)	52.5% Under $100 K (21)	53.2% Under $100 K (33)	*P* = 0.94
Caregiver race % (n)	55% White Non-Hispanic (22)	61.3% White Non-Hispanic (38)	*P* = 0.53
Caregiver time spent hrs/week mean (SD)	20.0 (33.6)	7.4 (17.0)	*P **< 0.01**
Caregiver duration yrs. mean (SD)	8.6 (10.3)	7.9 (7.3)	*P* = 0.85
Caregiver chronic disease % (n)	82.5% No (33)	88.7% No (55)	*P* = 0.38
Caregiver absenteeism %(n)	58.1% Yes (18)	23.8% Yes (10)	*P < 0.01
Caregiver absenteeism mean (SD)	14.1% (19.7)	5.4% (13.8)	**#*P*** **= 0.04**
Caregiver presenteeism %(n)	83.9% Yes (26)	37.2% Yes (16)	*P **< 0.01**
Caregiver presenteeism, *mean (SD)*	38.1% (33.9)	9.5% (20.3)	**#*P*** **< 0.01**
Caregiver activity impairment %(n)	80% Yes (32)	33.9% Yes (21)	*P **= 0.01**
Caregiver activity impairment *mean (SD)*	36.5% (30.3%)	7.3% (14.3%)	**#*P*** **< 0.01**

In the simple logistic regression models, 15 variables had a *p*-value of <.35 (Table 3). These variables were entered in a multiple regression model, which revealed that white non-Hispanic race (*p* = .02), the IBD patient having a history of a fistula (*p* = .01), a UC diagnosis (versus CD; *p* < .01), active disease (*p* < .01) and time spent on caregiving (*p* < .01) were independent predictors for caregiver burden (Table 4).

**Table 4:** Multivariate stepwise regression results for caregiver burden

Variable	Estimate	Standard Error	***p***-value
**Race** - White Non-Hispanic	1.4147	0.6037	0.02
**History of Fistula** - Yes	1.5534	0.6199	0.01
**IBD subtype** UC	1.7265	0.5946	< 0.01
**Active Disease** - Yes	1.6349	0.554	< 0.01
**Caregiver Education** - College or post-college	1.2586	0.6345	0.04
**Time spent on Caregiving** (hours)	0.5286	0.1452	< 0.01

## Discussion

This study reveals three important new insights for IBD patients and their caregivers: First, caregiving for IBD patient’s causes significant productivity decreases that have not been reported before, with absenteeism rates as high as 38% and presenteeism as high as 58% in caregivers who experience burden. Second, we report on new predictors for caregiver burden, including a UC diagnosis (versus CD) and a history of fistulas. Finally, despite the burden, caregivers feel they should be doing more for their care recipient and feel they could do a better job at caregiving, warranting the need for more caregiver solutions.

Prior literature has shown that IBD caregivers retire early, change from full-time to part-time positions, or face work termination due to caregiving responsibilities [19]. However, an evaluation of presenteeism and absenteeism in IBD caregivers has not been performed. Our study showed that caregivers with burden have significantly more absenteeism (58%) and presenteeism (84%) than caregivers without burden (24% absenteeism and 37% presenteeism). These reductions in work productivity might be explained by the number of hours required to care for an IBD patient, which is consistent with our findings that caregivers who spend more time with their care recipient are more likely to experience burden. Our group has previously shown the dramatic economic impact of decreased productivity in the working IBD population [4]; our findings suggest there also may be hidden costs associated with IBD caregiving.

It is known that intensive caregiving can affect caregivers mentally, physically, and economically [4, 12, 19]. While there are many publications about caregivers for other chronic diseases, the literature on IBD caregiving is scarce [5–7, 20]. A study on IBD caregivers of adult IBD patients by Parekh et al. showed comparable findings to ours. Similar to Parekh’s study, we found that active and more severe IBD disease are predictors for high caregiver burden. In contrast, their results suggest gender (female), age (younger), annual income level (less than $30,000), and a personal history of psychiatric illness also play a role in caregiver burden whereas our findings do not identify these factors as predictors. On the other hand, we found that caregivers who cared for a UC patient were more likely to experience caregiver burden than those who cared for a CD patient. It is possible that these differences are related to differences in the educational levels of the studies’ participants; in Parekh’s study a minority of patients had an education at the college level or above (30%) [9], compared to 78% in our population.

There are several limitations of our study. Due to an incomplete response rate our study may suffer from selection bias. The reasons for our low response rates are not clearly understood. We speculate that questionnaire fatigue played a role in both IBD patients and caregivers. Additionally, some IBD patients in clinic expressed they did not have a caregiver, or anyone aiding them that met our description. Furthermore, our results showed that the non-responder group (IBD patients that could not be matched to a caregiver) had worse disease outcomes, more employment and more activity impairment, this might have led to understated caregiver burden results. Furthermore, our study was a cross-sectional assessment and not a longitudinal one, because we assessed our outcomes at one point in time the effects of surgeries, hospitalizations, depression and anxiety on caregiver burden might be understated. Moreover, most of our participants were white non-Hispanic and were college-educated, which might affect the generalizability of our results to other populations. Lastly, due to the small sample size of this study, we were limited in exploring differences in outcomes based on stratification of our population on disease activity and medical therapy.

In summary, this study offers multiple new insights about caregiver burden to the existing IBD literature. First, caregiver absenteeism, presenteeism, and activity impairment are prevalent in IBD caregivers and these impairments are exacerbated when the IBD patient’s disease is active. Our study suggests that disease activity in IBD patients and productivity in their caregivers are intertwined. Caregivers of IBD patients with active disease experience more burden, and caregivers with burden experience significantly more absenteeism, presenteeism, and activity impairment than caregivers without burden. These findings suggest that caregiver burden could have a substantial impact on the overall indirect cost associated with IBD. Second, we identified predictors for caregiver burden that had not previously been identified, including a UC diagnosis (versus CD) and a history of fistulas. Lastly, we found that caregivers feel that they should be doing more for their care recipient and feel they could do a better job at caregiving.

Shulz and Quittner have pointed out that a care recipient’s poor QoL can negatively affect the caregiver’s QoL as well [21]. In order to combat this, Shukla et al. recommends physicians to be proactive in screening caregivers and offer professional mental support (i.e. psychologists), educational materials, and problem-focused advice [8]. This need is confirmed by our results which show that IBD caregivers felt stressed between caring for the care recipient and trying to meet other responsibilities for family or work (41%) and they experienced fear for the future of the care recipient (73%).

Examples of interventions found in the literature that can positively empower patients and their caregivers are web-based and in-person support groups, being around those who are alike seems to help patients and caregivers [22, 23]. Furthermore, behavioral interventions using web-based and mobile apps, have the power to provide accessibility to patients for better maintenance of their IBD, as well as motivation to engage in positive behavior [24], this could potentially apply to their caregivers as well.

## Conclusions

By giving IBD patients the necessary tools to become an active stakeholder and providing caregivers with the necessary education and social support, a cooperative role in disease management may be able to reduce caregiver burden and increase caregiver empowerment. These efforts might relieve the detrimental effects on caregiver work productivity and could combat the uncertainty caregivers currently experience with regards to their care recipient. More intervention studies implementing solutions in caregivers for IBD patients could give the much-needed answers to a frequently overseen problem in IBD caregivers.

## Supplementary information

**Additional file 1 **Supplementary **Table 1.** Comparison of responder/non-responder populations across patient features. t-test for continuous and chi-squared for binary.

## Data Availability

The data that support the findings of this study are available from UCLA but restrictions apply to the availability of these data, which were used under license for the current study, and so are not publicly available. Data are however available from the authors upon reasonable request and with permission of UCLA.

## Abbreviations

IBD: Inflammatory bowel diseases
CD: Crohn’s disease
UC: Ulcerative colitis
QoL: Quality of life
REDCap: Research electronic data capture
WPAI-IBD: Work productivity and activity impairment questionnaire for IBD
sIBDQ: Short-IBD questionnaire
mHI-UC: Mobile Health Index UC
mHI-CD: Mobile Health Index CD
WPAI-CG: Work productivity and activity impairment questionnaire for caregivers
ZBI: Zarit Burden interview score
UCLA IRB: University of California Los Angeles institutional review board
