# Supplementary material for: The effects of inflammatory bowel disease on caregivers: significant burden and loss of productivity
Source: BMC Health Serv Res. 2020 Jun 18;20:556. doi: 10.1186/s12913-020-05425-w (PMC7302133; doi:10.1186/s12913-020-05425-w)
Supplement: Supplementary file 1 — Additional file 1 Supplementary Table 1. Comparison of responder/non-responder populations across patient features. t-test for continuous and chi-squared for binary. [file 12913_2020_5425_MOESM1_ESM.docx]

**Supplementary Table 1: Patient feature t-test (continuous) and chi-squared(binary) comparisons between patients with and without caregiver information**

| Variable | 92 IBD  Non-Responder group  (No matching caregiver)  Mean/Prop | 102 IBD  Responder group  (matching caregiver)  Mean/Prop. | p-value |
| --- | --- | --- | --- |
| Patient Age | 36.372 | 41.209 | P=0.02 |
| Patient Gender Female | 0.435 | 0.696 | P<0.01 |
| Patient Race Other | 0.337 | 0.176 | P=0.02 |
| Surgery | 0.739 | 0.539 | P<0.01 |
| Fistula | 0.783 | 0.667 | P=0.10 |
| Activity Impairment | 0.869 | 0.539 | P<0.01 |
| Active Disease | 0.543 | 0.216 | P<0.01 |
| Employed | 0.815 | 0.510 | P<0.01 |
